# Supplementary material for: Dual mechanisms of ictal high frequency oscillations in human rhythmic onset seizures
Source: Sci Rep. 2020 Nov 5;10:19166. doi: 10.1038/s41598-020-76138-7 (PMC7645614; doi:10.1038/s41598-020-76138-7)
Supplement: Supplementary file 1 — Supplementary Information. [file 41598_2020_76138_MOESM1_ESM.docx]

**Supplementary Material: Dual mechanisms of ictal high frequency oscillations in human rhythmic onset seizures**

Elliot H. Smith, Edward M. Merricks, Jyun-You Liou, Camilla Casadei, Lucia Melloni, Thomas Thesen, Daniel Friedman, Werner Doyle, Ronald G. Emerson, Robert R. Goodman, Guy M. McKhann II, Sameer A. Sheth, John D. Rolston, Catherine A. Schevon

**Supplementary Figure 1. Different correlations in pre-recruitment and post-recruitment MUA firing rate and BHF power.** The panel on the left shows a scatter plot of BHF power and MUA firing rate for each discharge in the pre-recruitment period. The panel on the right shows a scatter plot of BHF power and MUA firing rate for each discharge in the post-recruitment period. The Pearson’s correlations and corresponding p-values are displayed above each corresponding plot.

**Supplementary Figure 2. Dominant Frequency Detection.** **a**, example broadband LFP from an ECoG electrode. **b**, spectrogram of the seizure recorded on the electrode shown in **a**. **c**, dominant frequencies for all recorded ECoG electrodes through the duration of the seizure. Each colored line represents the dominant frequency from a distinct ECoG electrode. Seizure onset and offset are indicated with arrows.

**Supplementary Figure 3. Permutation distribution for Kuiper tests for MUA-HFO coupling.** A histogram of test statistics from the shuffled Kuiper tests. The values of test statistics for three tests performed with the real data from three frequency bands are shown above the histogram.

**Supplementary Figure 4. Spatiotemporal profile of recruitment of a seizure in which the core is in the MTL.** **a**, example broadband LFP from an ECoG electrode that is not recruited into the ictal core, i.e. that remains in the penumbra. **b**, HFO amplitude from the electrode shown in **a** exhibiting large discharges just before recruitment (the time of recruitment is indicated with gray dotted line). **c**, A spectrogram of the seizure recorded on the electrode shown in **a**. **d**, example broadband LFP from an ECoG electrode that is rapidly recruited into the seizure core. **e**, HFO amplitude from the electrode shown in **d**. **f**, spectrogram of the seizure recorded on the electrode shown in **d**.

**Supplementary Table 1. Clinical characteristics of patients from whom seizures were examined.**

| **Patient number**  **(age/sex)** | **1 (32/F)** | **2 (29/M)** | **3 (19/F)** | **4 (35/F)** |
| --- | --- | --- | --- | --- |
| **Type of Implant (microelectrodes)** | ECoG (MEA) | ECoG (MEA) | sEEG (BF) | sEEG (BF) |
| **Microwire array implant location** | Left inferior temporal gyrus, 2.5 cm from | Anterior middle temporal gyrus | Mesial temporal lobe (amygdala & hippocampus) | Mesial temporal lobe (amygdala & hippocampus) |
| **Number of seizures examined** | 3 | 1 | 1 | 1 |
| **ILAE seizure class** | Focal onset with impaired awareness | Focal to bilateral tonic-clonic | Focal onset with impaired awareness | Focal onset with impaired awareness |
| **Clinically defined seizure onset zone** | Left basal anterior temporal | Right posterior temporal with secondary generalization | Left mesial temporal | Left mesial temporal |
| **Resection Site** | Left basal anterior temporal | Right anterior temporal with inferior and middle temporal gyrus | Laser ablation of left mesial temporal lobe | No resection: responsive neurostimulator |
| **Surgical outcome (follow-up at time of writing in months)** | Engel 1a (55) | Engel 1a (36) | Engel 1d (32.7) | Engle 2c (17.4) |
